# Control of *Drosophila* Blood Cell Activation via Toll Signaling in the Fat Body

**DOI:** 10.1371/journal.pone.0102568

**Published:** 2014-08-07

**Authors:** Martin R. Schmid, Ines Anderl, Laura Vesala, Leena-Maija Vanha-aho, Xiao-Juan Deng, Mika Rämet, Dan Hultmark

**Affiliations:** 1 Department of Molecular Biology, Umeå University, Umeå, Sweden; 2 Institute of Biomedical Technology (BioMediTech), University of Tampere, Tampere, Finland; 3 College of Animal Science, South China Agricultural University, Guangzhou, China; 4 Department of Pediatrics, Tampere University Hospital, Tampere, Finland; University of Cambridge, United Kingdom

## Abstract

The Toll signaling pathway, first discovered in *Drosophila*, has a well-established role in immune responses in insects as well as in mammals. In *Drosophila*, the Toll-dependent induction of antimicrobial peptide production has been intensely studied as a model for innate immune responses in general. Besides this humoral immune response, Toll signaling is also known to activate blood cells in a reaction that is similar to the cellular immune response to parasite infections, but the mechanisms of this response are poorly understood. Here we have studied this response in detail, and found that Toll signaling in several different tissues can activate a cellular immune defense, and that this response does not require Toll signaling in the blood cells themselves. Like in the humoral immune response, we show that Toll signaling in the fat body (analogous to the liver in vertebrates) is of major importance in the Toll-dependent activation of blood cells. However, this Toll-dependent mechanism of blood cell activation contributes very little to the immune response against the parasitoid wasp, *Leptopilina boulardi*, probably because the wasp is able to suppress Toll induction. Other redundant pathways may be more important in the defense against this pathogen.

## Introduction

The immune response in *Drosophila* has become a useful model to understand important aspects of innate immunity in other organisms, including humans [1]–[3]. In response to bacterial or fungal infections, the flies produce a set of antimicrobial peptides [4], which are secreted into the hemocoel from the fat body, the fly's equivalent of a liver. The mechanisms of this humoral response have been intensively investigated during the past decades [5] and two signaling pathways were found to be particularly important, the Toll and Imd pathways, serving as models for the responses to the human Toll-like and TNF receptors, respectively [6], [7].

Despite the recent progress in this field, the biological role of the Toll pathway in *Drosophila* immunity is still somewhat enigmatic. Toll signaling is specifically activated by the Lys-type peptidoglycans that are found in the cell walls of many Gram-positive bacteria, while the Imd pathway responds most vigorously to the DAP-type peptidoglycans that are typical of other Gram-positive bacteria and of most or all Gram-negatives. Surprisingly, this specificity of induction is not matched by a corresponding target specificity of the induced effector molecules [8]–[10] (see also discussion in [1]). In fact, the Imd pathway is sufficient to induce the entire complement of antibacterial and antifungal peptides [9]. In contrast, the known output of Toll signaling is more restricted and it is not specifically geared towards those bacteria that have Lys-type peptidoglycans. Drosomycin, a standard example of Toll-induced effector molecules, is for instance an antifungal peptide with no known activity against bacteria, regardless of peptidoglycan structure [11].

However, Toll signaling is most likely important also for other aspects of immunity, such as the cellular immune response. In response to various kinds of immunological challenge, circulating hemocytes (blood cells) in the *Drosophila* larva increase in number and engage in defense reactions such as phagocytosis or encapsulation. For example, infection of *Drosophila* larvae by the parasitoid wasp *Leptopilina boulardi* causes the main class of hemocytes, the plasmatocytes, to leave sessile compartments and go into circulation. Many of them differentiate into lamellocytes, which are big flat cells that form heavily melanized capsules around the parasite egg [12]–[15]. Constitutively active *Toll* mutants, such as *Toll^10b^* (*Tl^10b^*, also called *Tl^8^*) [16], generate a very similar phenotype, with mobilization of sessile hemocytes, increased numbers of circulating hemocytes, lamellocyte formation, and aggregation of hemocytes in melanotic nodules [17]–[19], often called “tumors” or “pseudotumors”. In agreement with a possible role of Toll signaling in the cellular immune defense, Sorrentino *et al*. [20] reported that loss-of-function mutants in the Toll signaling pathway have a reduced capacity to encapsulate wasp eggs.

We have now studied the role of Toll signaling in the cellular immune response in more detail. Surprisingly, our results show that Toll signaling in non-hemocyte tissues, in particular the fat body, is more important for the activation of a hemocyte response than Toll signaling in the hemocytes themselves.

## Materials and Methods

### Fly stocks used

The following tissue-specific driver stocks were used: the hemocyte-specific drivers *Hml^Δ^-Gal4* (*w^1118^*; *P{Hml-GAL4.Δ}2 P{wUAS-2xEGFP}AH2*) [21] or *He-Gal4* (*P{He-GAL4.Z}*) [13], or a combination of the two (genotype: *eater-GFP, msn-Cherry; Hml^Δ^-Gal4; He-Gal4/TM6, Tb*). We also used the fat body-specific driver *FB-Gal4* (*P{GAL4}fat*) [22], [23], the hemocyte + fat body-specific driver *Cg-Gal4* (*w^1118^; P{Cg-Gal4.A}2*) [24], or the midgut-specific *NP3064-Gal4* driver (y* w*; P{GawB}NP3064). The *UAS-GFP* insert was removed from the *FB-Gal4* stock by recombination, and the driver was then backcrossed six times to *w^1118^* before we used it. *Hml^Δ^-Gal4* was obtained from Sergey Sinenko, but all Gal4 lines can be obtained from the Bloomington Drosophila Stock Center at Indiana University, except *NP3064-Gal4*, which was obtained from the Drosophila Genetic Resource Center (DGRC) in Kyoto.

Although all drivers we used are well established in the literature, we checked their tissue specificity by crossing to a *UAS-GFP* reporter. For the most important drivers this is illustrated in the Fig. S1. The *FB-Gal4* driver shows strong expression in the fat body (Fig. S1 A), with ectopic expression in oenocytes, salivary glands and trachea, and weakly in anal pads, but there is no detectable expression in the hemocytes (Fig. S1 B). The tissue specificity is the same in the gain-of-function *Toll^10b^* mutant (Fig. S1 C, C′ and D) as in the wild-type background. The *Hml^Δ^-Gal4* driver is expressed in a majority of the hemocytes, but is down-regulated in the lamellocytes (Fig. S1 E–J). It shows no ectopic expression. In third instar larvae, the *He-Gal4* driver is expressed in about 80% of the hemocytes, including plasmatocytes, lamellocytes and crystal cells. There is also strong ectopic expression in salivary glands and weaker expression in parts of the midgut. Combining the two hemocyte drivers give expression in essentially all hemocytes. The *Cg-Gal4* driver is expressed in fat body and hemocytes, with no ectopic expression. The *NP3064-Gal4* driver is expressed in the midgut, and ectopically in salivary glands and posterior spiracles. At earlier stages this driver is also expressed elsewhere, including fat body and posterior hemocytes.

The binary *UAS-Gal4* system [25] was used to create specific loss-of-function phenotypes in larval intestines, hemocytes and/or fat body using fly lines carrying RNAi constructs for *Myd88* (*MyD88^GD25399^* and *MyD88^GD25402^*), *pelle* (*pll^GD2889^*), *dorsal* (*dl^GD45996^*), *Dif* (*Dif^GD30579^*) and *eater* (*ea^GD4301^*), all obtained from the Vienna Drosophila RNAi Center (VDRC). Control *w^1118^* iso flies, with the same isogenized genetic background [26], were obtained from the Szeged Drosophila Stock Center (now closed). The *UAS-Tl^10b^* stock (*y w P{UAS-Tl^10b^}11*), constructed by J.-M. Reichhart (Centre National de la Recherche Scientifique, Strasbourg, France), carries a *Toll^10b^* insert. Overexpression of this construct is known to activate the Toll pathway [13].

The constitutively active *Toll^10b^* mutant (*mwh^1^ e^1^ Tl^10b^/T(1;3)OR60/TM3, Sb^1^ Ser^1^*) [16] was obtained from the Bloomington Drosophila Stock Center at Indiana University. The following hemocyte class-specific reporter lines used in this study were obtained from Robert Schulz' lab: for plasmatocytes *eater-GFP* [27] and *eater-DsRed* [28], and for lamellocytes *MSNF9mo-mCherry* (hereafter called *msn-Cherry*) [28]. For *in vivo* observation of Toll activation, we used the *Drs-GFP* reporter (*P{Drs-GFP.JM804}1*) [29], obtained from D. Ferrandon.

### Fly crossing and handling of larvae

For each experimental cross 20 virgin females and 5–10 males of the desired genotypes were confined into a bottle containing standard potato food diet with yeast. Crosses were transferred into new bottles daily and kept in an incubator at 29°C and 60% humidity for optimal efficiency of the UAS/GAL4 system in their offspring. After 4-5 days larvae were staged according to procedures published elsewhere [30]. Wandering third instar larvae, at a stage just before the gut contents were completely cleared, were collected for *in vivo* inspection.

### Wasp infection

The genetic background of the fly stocks substantially influences the outcome of the wasp infection experiments. We therefore backcrossed all GAL4 driver lines six times to a *w^1118^* line. With this genetic background, approximately 50% of the larvae were successfully parasitized by *Leptopilina boulardi.* Gal4 driver virgin females were crossed to RNAi males. As controls served Gal4 driver virgins crossed to *w^1118^* iso males (the genetic background of the RNAi lines), and *w^1118^* virgin females (the genetic background of the Gal4 lines) crossed to RNAi construct males. This ensured that the genetic backgrounds of the experimental crosses were similar to those of the control crosses. The flies were kept at room temperature and transferred into fresh vials daily. The vials that contained the eggs were shifted to 29 °C. The fly larvae were infected by leaving them with twenty female and ten male wasps during two hours on the third day after egg lay. We used *L. boulardi G486* for all infection experiments. We scored encapsulation of wasp eggs 27–29 h and the ability to kill the parasitoid 48–50 h after infection. A wasp egg was counted as encapsulated if melanin had been deposited on it, and a parasite as successfully killed when we found melanized wasp eggs or melanized and killed wasp larvae within the body cavities of the dissected fly larvae. All experiments were done in triplicate and at least 100 infected larvae were scored for each experiment.

### Nodule frequency and grading of sessile hemocyte banding pattern

Before analysis, bottles containing the crosses were assigned with arbitrary numbers to blind the examiner and the real composition of the cross was not revealed before completion of the experiment. For assessing nodule frequencies, 50 F1 progeny third instar larvae from each cross were collected at random, gently washed with a paintbrush in water before being inspected for nodules under a standard stereo microscope. To grade the banded pattern of sessile hemocytes, additional larvae were collected and laid with their ventral side down on ice-cold glass slides. The larvae were then embedded in 50% ice-cold glycerol under a cover glass before being transferred into a refrigerator. After 20 min at −20°C or over-night incubation at +4°C, they were placed on ice and immediately analyzed under a fluorescence microscope. For each cross, 16–22 larvae were individually scored for the degree of disruption of the bands of sessile hemocytes under the epidermis [31]. A mobilization index was defined as follows: Grade 1, larvae with sessile hemocyte bands in all segments; Grade 2, and 3, bands of sessile cells in less than 75 and 50% of the segments respectively; Grade 4, no discrete band of sessile cells in any, or at most in the posterior 25% of the segments. All crosses were repeated three times and the nodule frequencies and the average mobilization indexes were calculated each time.

### Blood cell preparation and counting

To collect blood cells, 10 third instar larvae per cross were placed separately in the wells of a 12-well glass slide, each containing 20 µl of ice-cold phosphate-buffered saline (137 mM NaCl, 2.7 mM KCl, 6.7 mM Na_2_HPO_4_, 1.5 mM KH_2_PO_4_). The animals were carefully ripped open with the help of two watchmaker forceps, the carcasses were removed from the glass slide, and 10 µl of the blood cell suspension were transferred to a Neubauer hemocytometer chamber (Paul Marienfeld GmbH & Co. KG, Lauda-Königshofen, Germany). Lamellocytes and plasmatocytes were distinguished based on their morphology and counted in a phase contrast microscope (Axioplan, Carl Zeiss, 40–60 x magnification).

### *Drs-GFP* induction experiment

Twenty *Drs*-GFP females were crossed to ten *w^1118^* males. The crosses were treated as described above. 48–50 h after infection the fly larvae were first scored for *Drs-GFP* expression in the fat body and then for wasp infection. *Drs-GFP* was scored according to the strength of GFP induction as GFP^-^ (no GFP induction), GFP^+^ (weak GFP induction mainly in the posterior fat body) and GFP^++^ (strong and systemic GFP expression in the entire fat body). The infected fly larvae were then further divided into different categories, depending on the outcome of the infection. As described previously [29], a few individuals showed GFP expression in parts of the tracheal system. This phenotype was independent of wasp infection and was therefore not included in the analysis. All experiments were done in triplicate, with at least 200 infected larvae in each experiment. GFP expression was observed with a Nightsea add-on light & filter set (with Royal Blue color light head), attached to a standard dissection microscope.

### Phagocytosis assay with primary hemocytes

Larval hemocytes were isolated from the offspring of the indicated crosses. *Ex vivo* bacterial phagocytosis assays were performed as described earlier [32], with the following modifications: wandering third instar larvae were disinfected in 5% sodium hypochlorite solution for 2 min, washed 3 times in H_2_O, and bled into 1 ml ice-cold Schneider's *Drosophila* medium (Sigma-Aldrich). Then, excess medium was removed, 3×10^6^ FITC-labeled bacteria were added and centrifuged briefly onto the cells, and the cells were allowed to phagocytose for 10 min at 25 °C. Plates were returned on ice, the cells were fixed with 2% glutaraldehyde at room temperature, and extracellular fluorescent particles were quenched with a trypan blue solution. Microscopy and imaging were performed using Olympus IX71 microscope with F-view soft imaging system and QCapture Pro 6.0 software.

### Imaging and microscopy

Images were taken with a Nikon Digital sight color camera (Ds-Fi1), through a Nikon Eclipse 90i microscope run by NIS-Elements AR software. ApoTome images were taken with a Zeiss AxioCam (HRm) through a Zeiss AxioImager.M2 microscope with ApoTome2 for structured illumination. All images were enhanced using Photoshop CS3 software.

### Statistical analysis

A Kruskal-Wallis test was run to determine differences in the average mobilization index calculated for repeated crosses of the same genotype as well as different genotypes. Pairwise comparisons were performed using Dunn's procedure with a Bonferroni correction for multiple comparisons [33]. Post-hoc analysis was used to determine statistically significant differences of the average grades between crosses. Data from plasmatocyte counts was log_10_ transformed and then analyzed for significant differences using independent samples T-test (2-tailed), equal variances not assumed. Lamellocyte numbers of different crosses were compared using Mann-Whitney U exact test (2-tailed). The proportions of encapsulated wasp eggs and killed parasites of each cross were transformed by the arctangent function. Then, we used a 2-tailed T-test to compare the control crosses to the experimental crosses to determine statistical significance. All statistical data analysis was done with the IBM SPSS software, version 20.

## Results

### Tissue-specific activation of toll signaling

**Figure 1:**
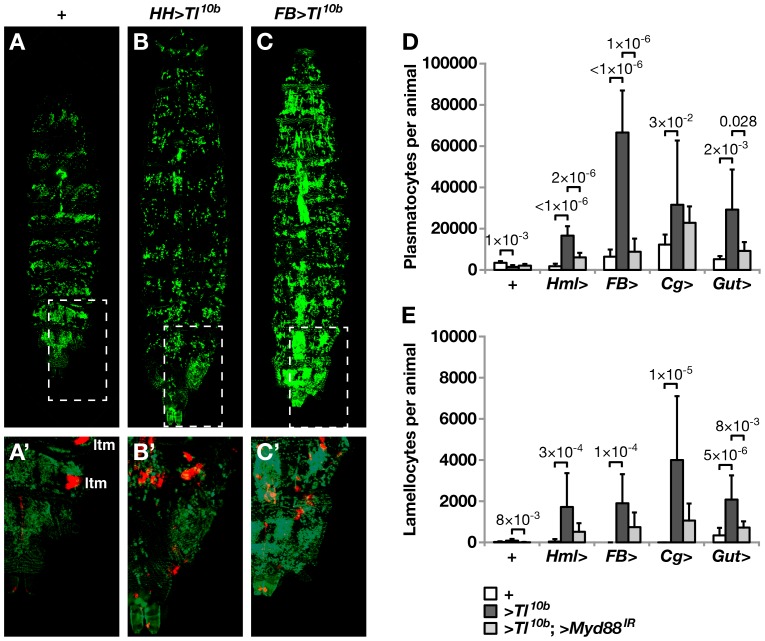
Forced Toll signaling in several different tissues triggers a hemocyte response. The pattern of sessile cells in a control third instar larva (**A**), or after expression of *UAS-Toll^10b^* (>*Tl^10b^*) in hemocytes with the *Hml^Δ^-Gal4*, *He-Gal4* (*HH*>) double driver (**B**) or in fat body with the *FB-Gal4* (*FB*>) driver (**C**). The total number of circulating plasmatocytes (**D**) and lamellocytes (**E**) is dramatically increased in the hemolymph of larvae when *UAS-Toll^10b^* (>*Tl^10b^*) is expressed with different drivers: in hemocytes by *Hml^Δ^-Gal4* (*Hml*>), in fat body by *FB-Gal4* (*FB*>), in hemocytes plus fat body by *Cg-Gal4* (*Cg*>), and in intestine by *NP^3084^-Gal4* (*Gut*>), compared to the *w^1118^* control (+). This effect was suppressed by co-expressing the *UAS-MyD88^GD25399^* RNAi construct (>*MyD88^IR^*). In A-C, plasmatocytes were visualized with the *eater-GFP* reporter, and in A′-C′ also lamellocytes with the *msn-Cherry* reporter, using ApoTome imaging. In the control larva (A′) *msn-Cherry* is ectopically expressed in the lateral transverse muscles (ltm). In the *Toll^10b^*-expressing larvae (B′, C′), *msn-Cherry* fluorescence is mainly seen in the lamellocytes. Larvae of the genotype shown in (C) had to be kept at 26 °C, all other experiments were at 29 °C. The larvae are oriented with the anterior end up. In D and E the cell types were distinguished by morphology and their numbers were counted in hemolymph samples of 10 individual larvae per cross. The results are presented as the means +/− standard deviation and the significance levels were estimated by independent sample T-test (two-tailed) in D and Mann-Whitney U exact test (two-tailed) in E.

In agreement with previous observations [13], [31], we found that it was sufficient to express a constitutively active *UAS-Tl^10b^* construct in hemocytes to mimic most aspects of an activated cellular immune response, including a disruption of the segmental pattern of sessile hemocytes in the body of *Drosophila* larvae, as detected with the plasmatocyte-specific *eater-GFP* reporter (Fig 1B, compare to 1A), an increased number of circulating plasmatocytes (Fig. 1D), and generation of lamellocytes (Fig. 1E). Lamellocytes were also detected *in vivo* with the *msn-Cherry* reporter (Fig. 1B′). No lamellocytes were detected in the control, only ectopic *msn-Cherry* expression in the lateral transverse muscles, (ltm, Fig. 1A′). A natural assumption would therefore be that the hemocyte phenotype of the *Toll^10b^* mutant is caused by Toll signaling in the hemocytes themselves. However, we found as strong, or even stronger, effects when we expressed the *UAS-Tl^10b^* construct in other tissues (Fig. 1C–E). Strikingly, compared to the control, the total number of circulating plasmatocytes increased more than ten-fold when we used the *FB-Gal4* driver to express *UAS-Tl^10b^* in the fat body, suggesting a proliferative response (Fig. 1D). Strong effects were also seen when we used the midgut-specific *NP^3084^-Gal4* driver, or the *Cg-Gal4* driver, which is expressed both in hemocytes and fat body (Fig. 1D). Toll activation in any of these tissues also led to the appearance of variable numbers of circulating lamellocytes (Fig. 1B′, C′, E). The quantification of lamellocytes was not entirely reliable, as many of them end up in melanotic nodules, but the presence of lamellocytes confirmed that the cellular immune response program had been activated. The sessile band disruption phenotype [31] as well as the increased hemocyte numbers (Fig. 1D–E) caused by the *UAS-Tl^10b^* constructs were suppressed when we co-expressed an RNAi construct for the *MyD88* gene, which acts downstream of *Toll* in the Toll pathway. This confirms the role of Toll signaling for these effects.

### Requirement of toll signaling in the fat body for toll-dependent activation of a hemocyte response

**Figure 2:**
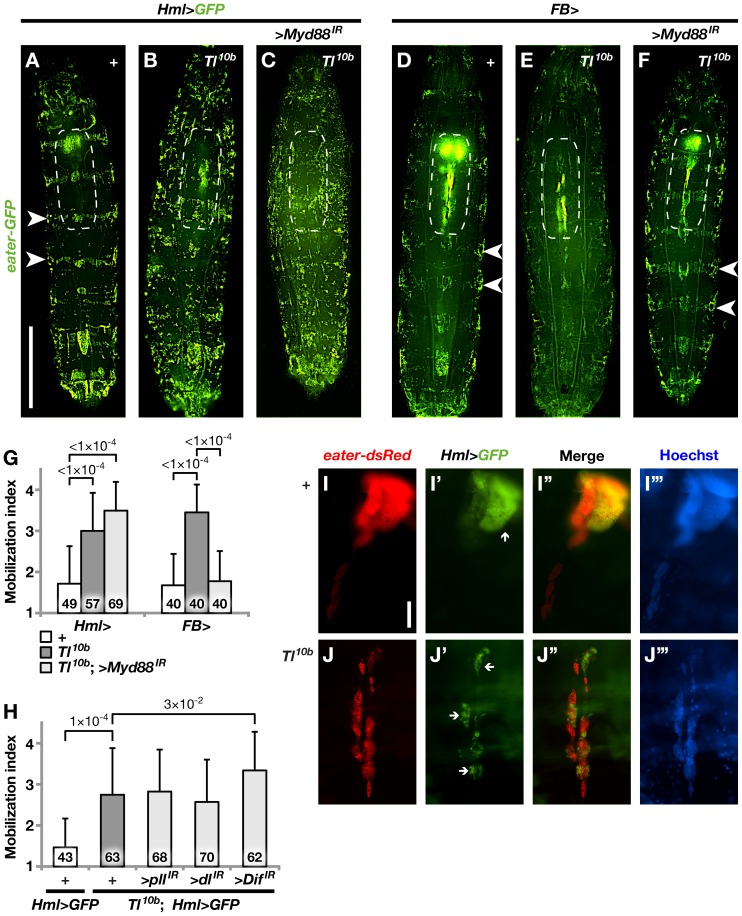
Mobilization of sessile hemocytes in the *Toll^10b^* gain-of-function mutant is suppressed after blocking Toll signaling in the fat body but not in the hemocytes. **A–F**. Plasmatocyte distribution is shown in control (+) and *Toll^10b^* gain-of-function mutant (*Tl^10b^*) third instar larvae, with or without suppression of Toll signaling by the *UAS-MyD88^GD25399^* construct (>*MyD88^IR^*), driven in hemocytes by *Hml^Δ^-Gal4* (*Hml*>) or in fat body by *FB-Gal4* (*FB*>). The plasmatocytes are visualized by *Hml^Δ^-Gal4*-driven *UAS-GFP* and the *eater-GFP* reporter (in A–C), or by *eater-GFP* alone (in D–F). Segmental bands of sessile plasmatocytes (white arrowheads) indicate that the plasmatocytes are in a resting state. Dashed lines demarcate the region occupied by the lymph glands. **G**. Quantification of the mobilization of sessile plasmatocytes in larvae of the genotypes shown in panel A. Mobilization index = 1 corresponds to a fully developed banding pattern and 4 indicates total loss of the sessile bands. The bars show the means +/− standard deviation, and sample sizes N are shown within the bars. **H**. Additional genotypes tested as in G. **I**, **J**. Morphology of dissected lymph glands from control (I–I′′′) or *Toll^10b^* mutant (J–J′′′) third instar larvae. Two plasmatocyte reporters are used to visualize the cells, *Hml^Δ^-Gal4*-driven *UAS-GFP*, which stains differentiated plasmatocytes in the lymph gland cortex, and *eater-DsRed*, which is also seen in the undifferentiated medullary cells. The larvae and lymph glands are oriented with the anterior end up. Size bar in A–F corresponds to 1 mm and in I–J to 0.1 mm.

Our results show that an activated Toll signaling pathway in tissues like the fat body is sufficient to cause an immune response-like phenotype in the hemocytes. The fat body is a major organ, especially in the larva, and the role of Toll signaling in this tissue is already well established in the context of humoral immunity. We therefore next investigated to what extent the fat body also contributes to the hemocyte phenotype of the *Toll^10b^* gain-of-function mutant. To observe hemocytes inside the living third-instar larvae we used three different fluorescent hemocyte markers: *Hml^Δ^-Gal4*-driven *UAS-GFP* (*Hml>GFP* for short) and/or *eater-GFP* for plasmatocytes, and *msn-Cherry* (*MSNF9mo-mCherry*) for lamellocytes [13], [28], [34]. As shown in Fig. 2, the segmental pattern of sessile plasmatocytes in control larvae (panels A and D, white arrowheads) was disrupted in the *Toll^10b^* mutant (panels B and E), like it was after *UAS-Tl^10b^* expression (Fig. 1B, C), indicating that the hemocytes had been mobilized. We could not suppress this *Toll^10b^* mutant phenotype by blocking Toll signaling in the hemocytes, using the *MyD88^GD25399^* RNAi construct, together with the hemocyte-specific *Hml^Δ^-Gal4* driver. If anything, it was even enhanced (panel C). In contrast, we could rescue the wild-type pattern by blocking Toll signaling in the fat body (panel F). These effects could be verified by quantitative scoring of the segmental pattern phenotype, and they were found to be highly significant (Fig. 2G). We know that *MyD88^GD25399^* is a reliable suppressor of Toll signaling [31]. In addition, we also tested to knock down other Toll pathway components in the hemocytes by expressing RNAi constructs for *pelle*, *dorsal* and *Dif*, but none of these constructs could suppress the *Toll^10b^* phenotype (Fig. 2H). Thus, we conclude that the mobilization of sessile hemocytes in the *Toll^10b^* mutant depends on Toll signaling in the fat body, but not in the hemocytes.

In wild-type larvae, plasmatocyte markers were mainly expressed in the large paired primary lobes of the lymph glands (Fig. 2I–I′′′; the lymph glands are also visible inside the demarcated regions in Fig. 2A and D). Within the primary lobes, *Hml*>*GFP* expression was restricted to the cortex. Unexpectedly, however, *eater-DsRed* was expressed in the entire lobe (Fig. 2I-I′′′) and, unlike *Hml*>*GFP*, *eater-DsRed* could also be detected in the secondary lobes. In the *Toll^10b^* mutant lymph glands we could observe a previously undescribed phenotype. The primary lobes appeared to be completely absent. Instead, rows of secondary lobes showed strong expression of both plasmatocyte markers (Fig. 2B and E, and Fig. 2J–J′′′). This phenotype must also be an indirect effect of Toll signaling in the fat body, as it was reversed when we blocked Toll signaling in the fat body (Fig. 2F), but not when we blocked Toll in the hemocytes (Fig. 2C).

**Figure 3:**
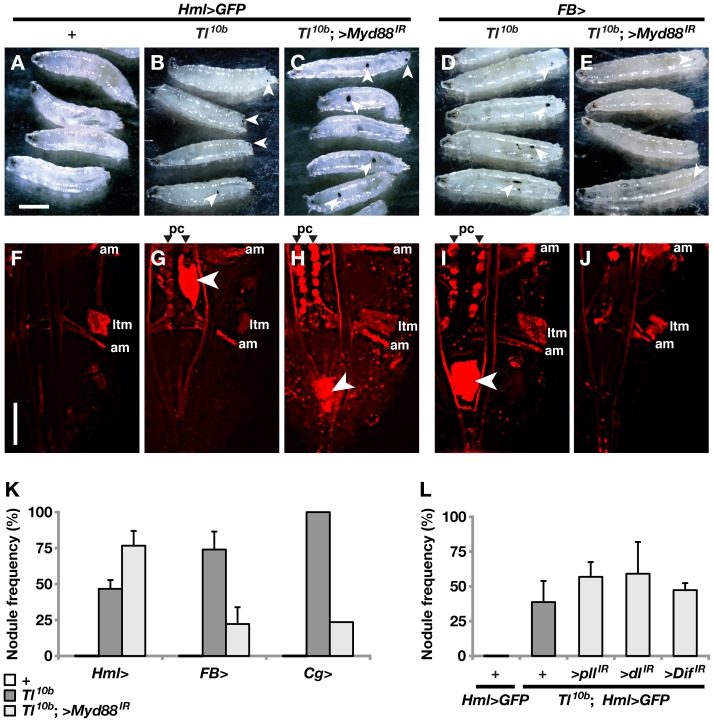
Toll-dependent formation of melanotic nodules requires Toll signaling in fat body but not in the hemocytes. **A–E**. Control (+) or *Toll^10b^* gain-of-function mutant (*Tl^10b^*) third instar larvae are shown with or without suppression of Toll signaling by the *UAS-MyD88^GD25399^* construct (>*MyD88^IR^*), driven in hemocytes by *Hml^Δ^-Gal4* (*Hml*>) or in fat body by *FB-Gal4* (*FB*>). Melanotic nodules are seen as black spots, as indicated by white arrowheads. **F–J**. Expression of the lamellocyte marker *msn-Cherry* in larvae of the same genotypes as in A–E. The posterior ends of the larvae are shown. Lamellocytes are incorporated in nodules (white arrowheads). Free lamellocytes can also be seen as small red dots in H and I. Strong ectopic expression of the *msn-Cherry* marker is seen in lateral transverse muscles (ltm) and alary muscles (am). In *Toll^10b^* mutants (G–I) it is also expressed in pericardial cells (pc), except when Toll is suppressed in the fat body (J). **K–L**. Bars represent the average frequency +/− standard deviation of larvae with at least one melanotic nodule, as calculated from three independent crosses for each of the genotypes described above, with 50 larvae in each experiment. In a single experiment, Toll signaling was simultaneously suppressed in hemocytes and fat body by the *Cg-Gal4* driver (*Cg*>). Size bars in A–E correspond to 1 mm and in F–J to 0.2 mm.

As a further sign of hemocyte activation in the *Toll^10b^* mutant, melanized hemocyte aggregates were seen in many of the mutant larvae (white arrowheads in Fig. 3B and D, quantified in Fig. 3K). These melanotic nodules became less frequent when we blocked Toll signaling in the fat body by expressing a *MyD88* RNAi construct (Fig. 3E and K). Furthermore the remaining nodules were smaller (Fig. 3E). In contrast, the size of and frequency of nodules increased when we blocked Toll signaling in the hemocytes (Fig. 3C and K). Expressing RNAi constructs that target other known Toll signaling components in hemocytes had a similar effect (Fig. 3L). *MyD88* knock-down in both fat body and hemocytes (by *Cg-Gal4*) did not further enhance the suppression of nodule frequencies compared to *MyD88* RNAi in fat body alone (Fig. 3K). These results give further support to the conclusion that signaling from fat body, not hemocytes, causes blood cell activation phenotypes in *Toll^10b^* mutant larvae.

The aggregates of hemocytes observed in the *Toll^10b^* mutant larvae were not all melanized, but they always included many lamellocytes, as shown by the strong expression of the marker for this cell type, *msn-Cherry* (Fig. 3G and I, white arrowhead). Strong ectopic expression of this marker in certain groups of muscles, such as the lateral transverse bundles of segmental muscles and the alary muscles of the heart (marked ltm and am in Fig. 3), made it difficult to follow the lamellocytes *in vivo*. However, scattered sessile lamellocytes were seen in the *Toll^10b^* mutant larvae (Fig. 3G and I). This phenotype was suppressed when we expressed a *MyD88* RNAi construct in the fat body (Fig. 3J), but enhanced when it was expressed in hemocytes (Fig. 3H). Furthermore, the *msn-Cherry* marker became activated in pericardial cells of the *Toll^10b^* mutant (marked pc in Fig. 3G and I), and this is also a phenotype that was suppressed when we blocked Toll signaling in the fat body (Fig. 3J), but enhanced when we blocked it in hemocytes (Fig. 3H).

**Figure 4:**
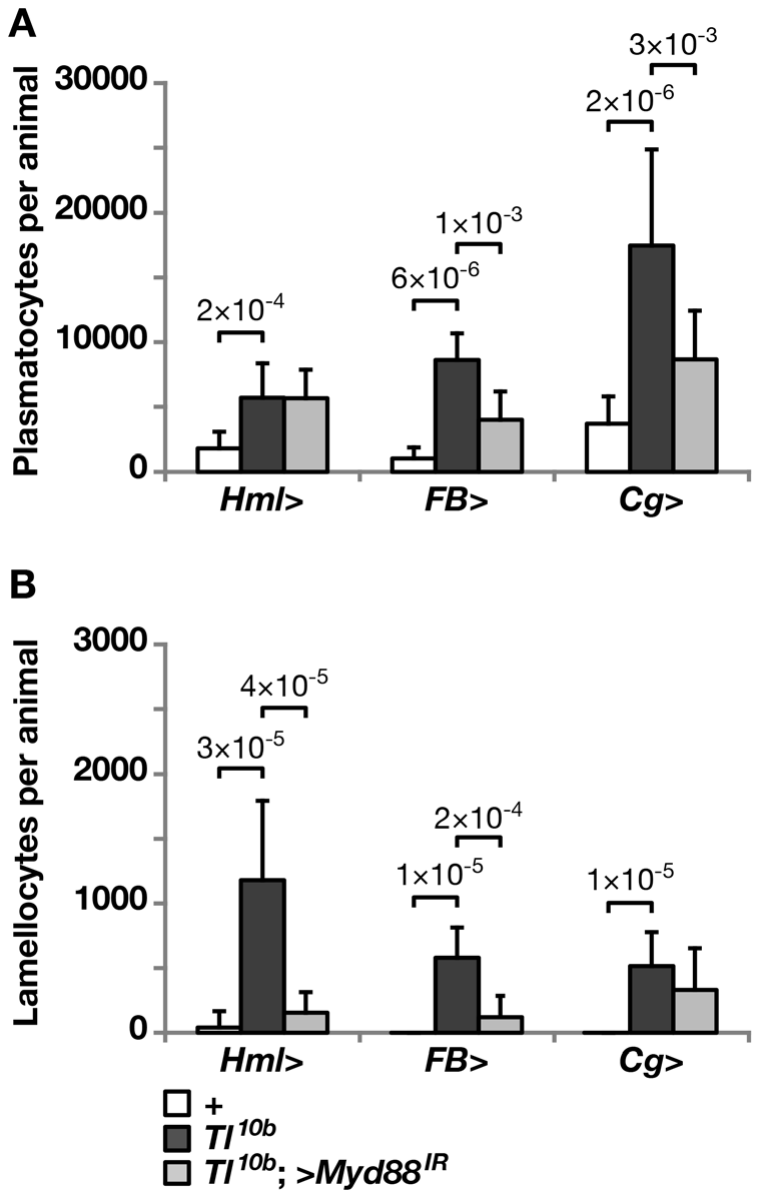
Toll-dependent hemocyte proliferation requires Toll signaling in fat body but not in hemocytes; Lamellocyte formation depends on Toll signaling in both tissues. **A**. The number of plasmatocytes is increased in the hemolymph of larvae carrying the *Toll* gain-of-function mutant *Toll^10b^*. This phenotype is not affected when Toll signaling is suppressed by the *UAS-MyD88^GD25399^* construct (>*MyD88^IR^*) with a hemocyte driver (*Hml*>), but it is partially reversed with fat body (FB>) or hemocytes plus fat body (*Cg*>) drivers. **B**. Circulating lamellocytes are found in *Toll^10b^* larvae. This phenotype is partially reversed when Toll signaling is suppressed by *Myd88* RNAi in hemocytes (*Hml*>), fat body (*FB*>) or in both hemocytes and fat body (*Cg*>). Plasmatocytes and lamellocytes were distinguished by morphology and counted separately. The results are presented as the means +/− standard deviation from three independent experiments with ten individual larvae in each cross, and the significance levels were estimated by independent sample T-test (two-tailed) in A and Mann-Whitney U exact test (two-tailed) in B.

The *Toll^10b^* mutant had increased numbers of plasmatocytes and lamellocytes in circulation (Fig. 4), although the increase was not as large as when we overexpressed a *UAS-Tl^10b^* construct in different tissues (Fig. 1). The plasmatocyte numbers were also affected by the different genetic backgrounds of the tested driver constructs (compare the dark bars in Fig. 4A), calling for some caution in the interpretation of our results. In line with our observations of other hemocyte-related phenotypes of the *Toll^10b^* mutant, the plasmatocyte numbers were reduced when we blocked Toll signaling in the fat body, but they were unaffected when Toll was blocked in hemocytes (Fig. 4A). Simultaneous expression of a *Myd88* RNAi construct in fat body and hemocytes had the same effect as expression in fat body alone. The number of lamellocytes was reduced, not only when we blocked Toll signaling in the fat body but also when we blocked Toll in hemocytes, or in both places (Fig. 4B). This was unexpected, as other aspects of the *Toll^10b^* phenotype become enhanced when Toll is blocked in the hemocytes. This paradox might be resolved by the observation that these larvae have bigger and more frequent nodules (Fig. 3C and K). It is therefore possible that the accumulation of lamellocytes in the nodules explains the reduced number of lamellocytes in circulation. Alternatively, we may have underestimated the number of lamellocytes if their morphology is not fully developed in these animals.

### The role of toll in the cellular immune defense

**Figure 5:**
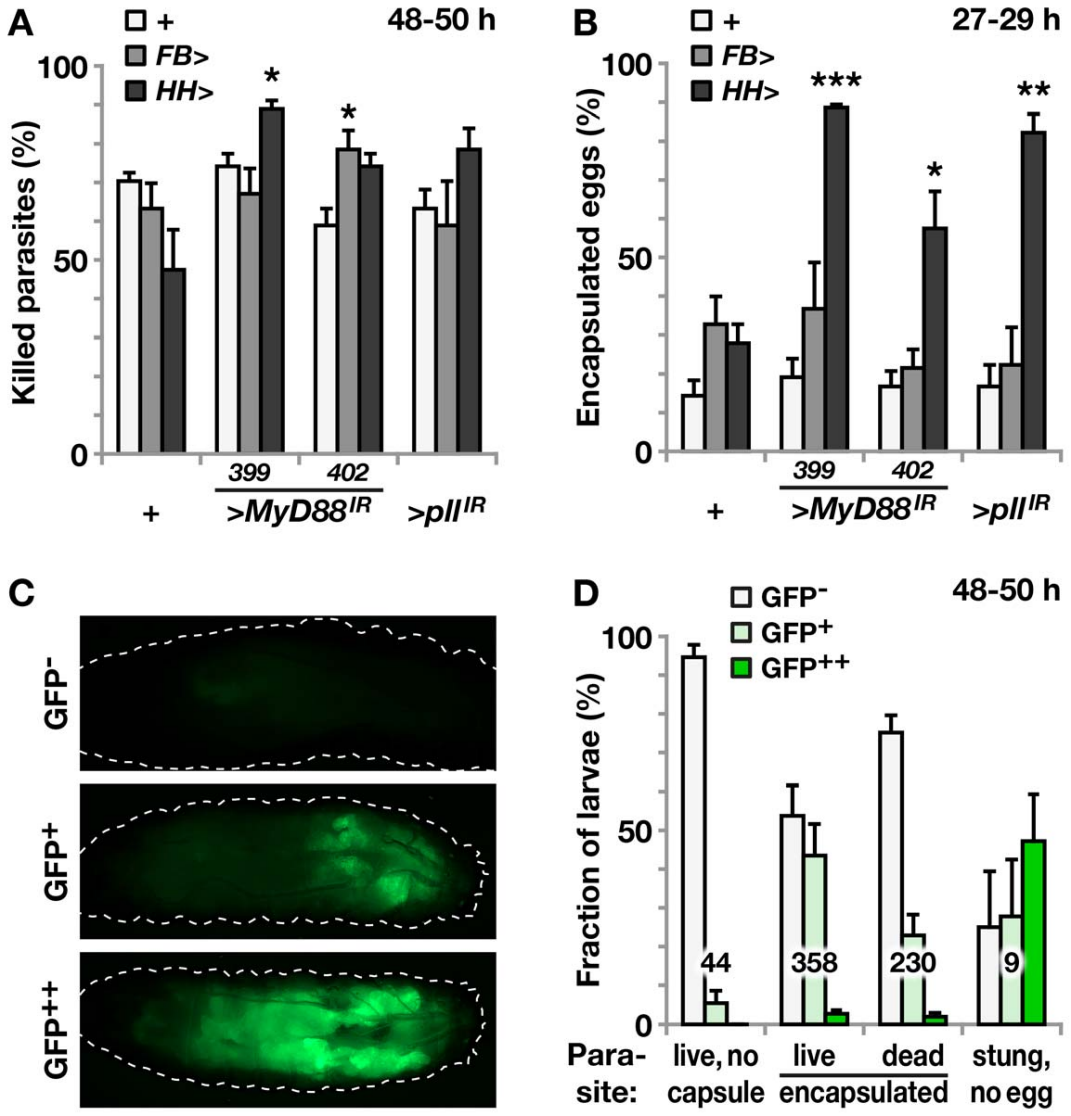
Killing of parasitoid wasp eggs does not require Toll signaling in the fat body or hemocytes, but the outcome of infection is correlated to Toll activation in the fat body. **A**. Percentage of killed parasites, as seen in dissected larvae 48–50 hours after infection. No consistent effect is seen after suppressing Toll signaling with the fat body driver *FB-Gal4* (*FB*>) or with the double hemocyte driver *Hml^Δ^-Gal4 He-Gal4* (*HH*>), crossed to *MyD88* RNAi constructs (>*MyD88^IR^*) *MyD88^GD25399^* (399) or *MyD88^GD25402^* (402), or to a *pelle* RNAi construct (>*pll^IR^*), *pll^GD2889^*. **B**. The percentage of parasitized larvae that have visible black capsules 27–29 hours after infection (genotypes are the same as in A). Suppressing Toll signaling with the double hemocyte driver *Hml-Gal4 He-Gal4* (*HH*>) does significantly enhance the encapsulation response, whereas suppressing Toll signaling with the fat body driver *FB-Gal4* (*FB*>) does not have any effect on encapsulation. **C**. Activation of Toll signaling in the fat body of parasitized larvae, as visualized with the *Drs-GFP* reporter and ApoTome imaging. Most larvae show no activation (GFP^-^) 48–50 hours after infection, some show weak activation, mainly in the posterior end (GFP^+^), and a few show strong generalized activation (GFP^++^). **D**. Correlation between Drs-GFP activation and the encapsulation response, as seen after dissecting larvae 48–50 hours after infection. The dissected larvae were divided into four categories, depending on the outcome of the infection: “live, no capsule” (living wasp larvae and no melanization), “live encapsulated” (living wasp larvae and destroyed melanized capsules), “dead encapsulated” (dead, melanized wasp larvae or melanized wasp eggs), and “stung, no egg” (fly larvae with a clear ovipositor wound but without a parasite). The bars show the percentage of larvae within each category with no, weak, or strong GFP expression. Average +/− standard error of the mean, from three independent experiments with more than 200 larvae in each experiment, * *p*<0.05, ** *p*<0.01, *** *p*<0.001. The total number of larvae in each category is indicated.

To test how Toll signaling affects the cellular immune defense, we followed the outcome of *Leptopilina boulardi* infections after suppressing Toll signaling by expressing RNAi constructs for *MyD88* or *pelle* in fat body or hemocytes. Contrary to our expectations, this had no consistent effect on the actual killing of the parasite, as scored in dissected larvae 48–50 h after infection, regardless of whether we blocked Toll in the fat body or hemocytes (Fig. 5A). We also scored the presence of melanized capsules at an earlier time point, 27–29 h after infection, without checking the survival of the parasites (Fig. 5B), but again we saw no significant effect when we blocked Toll signaling in the fat body. With the RNAi approach we cannot completely rule out that residual activity of the pathway may account for the encapsulation and killing of the wasp larvae, but the efficacy of the *MyD88* RNAi constructs was proven in the suppression of the *Toll^10b^* phenotype. *MyD88^GD25399^* has also previously been used successfully to block Toll signaling [31]. Surprisingly, the response was even enhanced when we blocked Toll in hemocytes. The latter finding was in line with the enhancement of the *Toll^10b^* melanotic nodule phenotype when Toll signaling was blocked in hemocytes (Fig. 3), but it gave no support for a positive role of Toll in the defense against this wasp, and we conclude that the encapsulation and killing of the wasp larvae must primarily rely on Toll-independent mechanisms.

Parasites tend to have mechanisms to suppress the immune defenses of their hosts. We therefore investigated the level of Toll activation during the course of infection, using larvae with a *Drs-GFP* construct as a reporter for Toll activity. In a majority of the infected larvae we were unable to detect any Toll activity (Fig 5C, GFP^-^). Of a total of 632 infected larvae, only 224 (35%) showed signs of Toll activation, in most cases manifested as a weak activity in the posterior fat body (Fig 5C, GFP^+^), although strong activation throughout the fat body was seen in a few cases (Fig. 5C, GFP^++^). By contrast, out of nine larvae that the wasp had stung without injecting any egg, Toll was activated in seven, and as many as four of them were scored as strong. Thus, the presence of a wasp egg was correlated to a reduced Toll response, giving support for a Toll suppressor being injected with the wasp egg. No GFP expression was seen in the fat body of control larvae that had not been exposed to wasps.

In spite of the negative findings described above, the immune response seemed to be correlated to the level of Toll activation. We dissected the larvae 48 h after infection and noted the outcome of the infection. Those larvae where the parasite was found alive and without signs of encapsulation (the “live, no capsule” category) were almost all GFP-negative (Fig. 5D). In contrast, Toll signaling was evident in almost half of the individuals where parasites were encapsulated but still alive (the “live, encapsulated” category). In the final category, where the parasite was encapsulated and killed (“dead, encapsulated”), the proportion of larvae that showed Toll activation was again smaller. Thus, the level of induction of Toll in the fat body is correlated to the level of the immune response.

We also investigated the possible influence of Toll signaling on another aspect of the cellular immune response, the phagocytosis of bacteria. Hemocytes from wandering third instar larvae were incubated *ex vivo* with fluorescently labeled *Escherichia coli* bacteria. However, with this assay we could not observe any effect of Toll up- or down-regulation, neither in the fat body nor in the hemocytes (Fig. S2).

## Discussion

Clearly, Toll signaling is involved in several immunity-related phenomena in *Drosophila*. The well-known humoral response, with the fat body as a main player, is largely cell-autonomous and the same thing is likely true for the local induction of antimicrobial peptides in hemocytes and other tissues. Our results underscore that Toll signaling can also be an important factor in the activation of a cellular immune response. However, as we have shown here, Toll-dependent activation of hemocytes is not a cell-autonomous phenomenon. Toll signaling in any one of several tested tissues, including hemocytes, is sufficient to trigger a hemocyte response. Surprisingly, however, the Toll pathway is not required in the hemocytes themselves, at least not in the context of a generalized Toll activation, like in the *Toll^10b^* gain-of-function mutant. In fact, the *Toll^10b^* phenotype is even enhanced when Toll signaling is suppressed in the hemocytes, indicating a possible negative feedback loop. Similarly, the encapsulation of parasitoid wasp eggs is more vigorous when Toll signaling is blocked in the hemocytes.

Like in the humoral immune response, we have shown that the fat body is a major player in Toll-dependent hemocyte activation. The fat body is a dominating organ in most insects, with a function similar to that of the liver in vertebrates. It is biosynthetically very active and a source of many components of the body fluids in *Drosophila*, the hemolymph. Toll induction in response to bacterial infection, as detected with the *Drs-GFP* reporter [29], is most strikingly seen in the fat body, and it is also in this tissue that we see a Toll response after wasp infection. Toll induction in the fat body of *L. boulardi*-infected larvae was also reported by Schlenke *et al*. [35]. Although other tissues may contribute, the relative importance of the fat body for Toll-dependent hemocyte activation is demonstrated by the strong suppression of the *Toll^10b^*-induced hemocyte phenotypes when we blocked Toll signaling in the fat body.

Besides the effects on circulating and sessile hemocytes that we have shown here, Toll signaling in the fat body also appears to feed back on the hematopoietic tissue. The constitutively active *Toll^10b^* mutation showed strong phenotypic effects on the lymph glands, an effect that went unnoticed in earlier work [19], [36]. The primary lobes were absent, perhaps prematurely disrupted or not properly formed, and instead the secondary lobes were hypertrophied. It was sufficient to suppress Toll signaling in the fat body to restore the wild-type phenotype. Thus, in a Toll-dependent way, the fat body can control several different aspects of hemocyte activation and differentiation.

We conclude that Toll signaling, particularly in the fat body, can act as a potent activator of a hemocyte response. It was therefore a surprise to find that this Toll-dependent activation of the hemocytes plays only a minor role, if any, in the immune defense against *L. boulardi*. A likely explanation is that wasps inject an inhibitor of Toll activation during oviposition. This idea is supported by our observation that the Toll response is attenuated in the infected *Drosophila* larvae, compared to individuals that were only poked with the ovipositor. The hypothetical inhibitor must act upstream of Toll itself, as wasp infection does not attenuate the constitutively activated expression of the *Drs-GFP* reporter in the *Toll^10b^* mutant ([35], and data not shown). One possible candidate for this inhibitor is the serpin described by Colinet *et al*. from the venom of *L. boulardi* [37]. During oviposition, the wasp injects this serine protease inhibitor into the host where it inhibits the enzymes that activate phenol oxidase, thereby blocking the melanization reaction. Since similar serine protease cascades are required to activate the Toll ligand Spätzle, it is possible that Toll signaling is blocked as well. A similar strategy has been found in some Ichneumonid wasps that parasitize lepidopteran hosts. During oviposition these wasps transfer symbiotic ichnoviruses that express vankyrins, IκB-like molecules that act as Toll pathway inhibitors when tested in *Drosophila* [38].

The observed correlation between Toll activation in the fat body and the outcome of the wasp infection suggests that Toll signaling does contribute to the defense. For instance, a minority of the infected *Drosophila* larvae failed completely to mount an immune response, and those larvae hardly ever showed any sign of Toll activation. However, under our conditions, the contribution of Toll signaling to the immune defense was not sufficient to influence the fate of the main population of host larvae to a significant extent. Working with classical mutations in the Toll pathway, Sorrentino *et al*. [20] could detect an effect, albeit modest, on the resistance against the wasps, but their experiments are difficult to compare with ours. First, the classical mutants might affect early stages of hematopoiesis in a way that our RNAi approach does not. The classical mutants also affect tissues that we have not tested, like for instance the posterior signaling center, where Toll signaling has been shown to affect hematopoiesis [38]. Second, the encapsulation response is extremely sensitive to genetic background, a factor that was easier to control in our crosses. In any case, it is clear that *Drosophila* must have other, Toll-independent, ways to activate the cellular defense against parasitoid wasps, and that the system is likely to be highly redundant.

Important questions that remain for the future are how the fat body communicates with the hemocytes, and how the fat body is activated in the first place. We have considered the possibility that the activated hemocyte phenotype reflects an autoimmune response against the fat body, which often dissociates earlier in *Toll^10b^* larvae than in the wild-type control ([18]; see also Fig. S1, panel C′). However, this explanation is unlikely since the melanotic nodules were not specifically associated with the fat body. The effects we observed on the lymph glands and pericardial cells also argue for a diffusible signal.

In the larva, the Toll ligand Spätzle is mainly produced by hemocytes [39]. This opens a possibility that the hemocytes are directly involved in the activation of the fat body [40], thus generating a positive feedback loop. However, Spätzle itself must then first be activated by proteolytic cleavage, which would require additional signals. A primary recognition event at the injected egg is not necessarily required to activate the fat body, as we saw a good Toll response in larvae that the wasp had stung without laying any egg. It is possible that signals are sent from the wound site. Alternatively, Toll might be activated by bacteria that are introduced via the wound. The antimicrobial immune response, triggered by bacterial infections and perhaps wounds, may act as a danger signal and boost a general arousal of the cellular defenses. Similarly, Parisi et al. [41] recently described an interaction between hemocytes, the Toll-activated fat body, and epithelial tumors, eventually leading to tumor cell death. These phenomena suggest that the fat body, and perhaps other tissues too, participate in a systemic response that controls the general activity level of the organism's defense systems.

## Supporting Information

Figure S1 Tissue specificity of the *FB* and *Hml* drivers. The expression of the *FB-Gal4* (**A–D**) and *Hml^Δ^-Gal4* (**E–J**) drivers was visualized by GFP fluorescence after crossing to the *UAS-GFP* reporter. Similar patterns were seen in *Toll* wild-type (A–B, E–G) and *Toll^10b^* genetic background (C–D, H–J). Panels A, C, C′, E and H show whole-body images of the GFP fluorescence. The demarcated areas in E and H are shown enlarged in panels F and I (bright-field images to the right). The fat body morphology in many, but not all *Toll^10b^* larvae is partially disrupted (compare C and C′). Panels B, D, G and J show hemolymph samples, in each case visualized by GFP fluorescence (GFP), Hoechst fluorescence (H) and differential interference contrast (DIC). Lamellocytes are marked by white arrowheads.
(PDF)

Figure S2 No effect of Toll signaling on the phagocytosis of bacteria. Toll signaling was activated by expression of *UAS-Toll^10b^* (>*Tl^10b^*), or suppressed by expression of *UAS-MyD88^GD25399^* (>*MyD88^IR^*), either in hemocytes by *Hml^Δ^-Gal4* (*Hml*>), or in fat body by *FB-Gal4* (*FB*>). As a control, the *eater* RNAi construct *ea^GD4301^* (>*eater^IR^*) was also tested, but gave little effect. Hemocytes from larvae with these genotypes were incubated with FITC-labeled *E. coli*, and the phagocytosed bacteria are visualized by fluorescence, after quenching of extracellular bacteria with trypan blue. The top rows show controls without drivers. The leftmost panels show the drivers alone.
(PDF)

## References

[1] Hultmark D (2003) *Drosophila* immunity: paths and patterns. Curr Opin Immunol 15: 12–19. 12495727 10.1016/s0952-7915(02)00005-5

[2] Lemaitre B, Hoffmann J (2007) The host defense of *Drosophila melanogaster* . Annu Rev Immunol 25: 697–743. 17201680 10.1146/annurev.immunol.25.022106.141615

[3] Fauvarque MO, Williams MJ (2011) *Drosophila* cellular immunity: a story of migration and adhesion. J Cell Sci 124: 1373–1382. 21502134 10.1242/jcs.064592

[4] Bulet P, Stöcklin R (2005) Insect antimicrobial peptides: structures, properties and gene regulation. Protein Pept Lett 12: 3–11. 15638797 10.2174/0929866053406011

[5] Imler JL (2014) Overview of *Drosophila* immunity: A historical perspective. Dev Comp Immunol 42: 3–15. 24012863 10.1016/j.dci.2013.08.018

[6] Lindsay SA, Wasserman SA (2014) Conventional and non-conventional *Drosophila* Toll signaling. Dev Comp Immunol 42: 16–24. 23632253 10.1016/j.dci.2013.04.011 PMC3787077

[7] Kleino A, Silverman N (2014) The *Drosophila* IMD pathway in the activation of the humoral immune response. Dev Comp Immunol 42: 25–35. 23721820 10.1016/j.dci.2013.05.014 PMC3808521

[8] Lemaitre B, Nicolas E, Michaut L, Reichhart J-M, Hoffmann JA (1996) The dorsoventral regulatory gene cassette *spätzle*/*Toll*/*cactus* controls the potent antifungal response in Drosophila adults. Cell 86: 973–983. 8808632 10.1016/s0092-8674(00)80172-5

[9] Hedengren M, Åsling B, Dushay MS, Ando I, Ekengren S, et al (1999) *Relish*, a central factor in the control of humoral, but not cellular immunity in *Drosophila* . Mol Cell 4: 827–837. 10619029 10.1016/s1097-2765(00)80392-5

[10] De Gregorio E, Spellman PT, Tzou P, Rubin GM, Lemaitre B (2002) The Toll and Imd pathways are the major regulators of the immune response in *Drosophila* . EMBO J 21: 2568–2579. 12032070 10.1093/emboj/21.11.2568 PMC126042

[11] Fehlbaum P, Bulet P, Michaut L, Lagueux M, Broekaert WF, et al (1994) Insect immunity - septic injury of Drosophila induces the synthesis of a potent antifungal peptide with sequence homology to plant antifungal peptides. J Biol Chem 269: 33159–33163. 7806546

[12] Rizki TM, Rizki RM (1992) Lamellocyte differentiation in *Drosophila* larvae parasitized by *Leptopilina* . Dev Comp Immunol 16: 103–110. 1499832 10.1016/0145-305x(92)90011-z

[13] Zettervall CJ, Anderl I, Williams MJ, Palmer R, Kurucz E, et al (2004) A directed screen for genes involved in *Drosophila* blood cell activation. Proc Natl Acad Sci USA 101: 14192–14197. 15381778 10.1073/pnas.0403789101 PMC521135

[14] Márkus R, Laurinyecz B, Kurucz É, Honti V, Bajusz I, et al (2009) Sessile hemocytes as a hematopoietic compartment in *Drosophila melanogaster* . Proc Natl Acad Sci USA 106: 4805–4809. 19261847 10.1073/pnas.0801766106 PMC2660760

[15] Honti V, Csordás G, Márkus R, Kurucz É, Jankovics F, et al (2010) Cell lineage tracing reveals the plasticity of the hemocyte lineages and of the hematopoietic compartments in *Drosophila melanogaster* . Mol Immunol 47: 1997–2004. 20483458 10.1016/j.molimm.2010.04.017

[16] Erdelyi M, Szabad J (1989) Isolation and characterization of dominant female sterile mutations of *Drosophila melanogaster*. I. Mutations on the third chromosome. Genetics 122: 111–127. 2499514 10.1093/genetics/122.1.111 PMC1203676

[17] Gerttula S, Jin Y, Anderson KV (1988) Zygotic expression and activity of the Drosophila *Toll* gene, a gene required maternally for embryonic dorsal-ventral pattern formation. Genetics 119: 123–133. 2456252 10.1093/genetics/119.1.123 PMC1203330

[18] Lemaitre B, Meister M, Govind S, Georgel P, Steward R, et al (1995) Functional analysis and regulation of nuclear import of *dorsal* during the immune response in *Drosophila* . EMBO J 14: 536–545. 7859742 10.1002/j.1460-2075.1995.tb07029.x PMC398111

[19] Qiu P, Pan PC, Govind S (1998) A role for the *Drosophila* Toll/Cactus pathway in larval hematopoiesis. Development 125: 1909–1920. 9550723 10.1242/dev.125.10.1909

[20] Sorrentino RP, Melk JP, Govind S (2004) Genetic analysis of contributions of dorsal group and JAK-Stat92E pathway genes to larval hemocyte concentration and the egg encapsulation response in Drosophila. Genetics 166: 1343–1356. 15082553 10.1534/genetics.166.3.1343 PMC1470785

[21] Sinenko SA, Mathey-Prevot B (2004) Increased expression of *Drosophila* tetraspanin, Tsp68C, suppresses the abnormal proliferation of ytr-deficient and Ras/Raf-activated hemocytes. Oncogene 23: 9120–9128. 15480416 10.1038/sj.onc.1208156

[22] Grönke S, Beller M, Fellert S, Ramakrishnan H, Jäckle H, et al (2003) Control of fat storage by a *Drosophila* PAT domain protein. Curr Biol 13: 603–606. 12676093 10.1016/s0960-9822(03)00175-1

[23] Maxton-Küchenmeister J, Handel K, Schmidt-Ott U, Roth S, Jäckle H (1999) *Toll* homologue expression in the beetle *Tribolium* suggests a different mode of dorsoventral patterning than in *Drosophila* embryos. Mech Dev 83: 107–114. 10381571 10.1016/s0925-4773(99)00041-6

[24] Asha H, Nagy I, Kovacs G, Stetson D, Ando I, et al (2003) Analysis of Ras-induced overproliferation in Drosophila hemocytes. Genetics 163: 203–215. 12586708 10.1093/genetics/163.1.203 PMC1462399

[25] Brand AH, Perrimon N (1993) Targeted gene expression as a means of altering cell fates and generating dominant phenotypes. Development 118: 401–415. 8223268 10.1242/dev.118.2.401

[26] Ryder E, Blows F, Ashburner M, Bautista-Llacer R, Coulson D, et al (2004) The DrosDel collection: a set of *P*-element insertions for generating custom chromosomal aberrations in *Drosophila melanogaster* . Genetics 167: 797–813. 15238529 10.1534/genetics.104.026658 PMC1470913

[27] Sorrentino RP, Tokusumi T, Schulz RA (2007) The Friend of GATA protein U-shaped functions as a hematopoietic tumor suppressor in *Drosophila* . Dev Biol 311: 311–323. 17936744 10.1016/j.ydbio.2007.08.011

[28] Tokusumi T, Shoue DA, Tokusumi Y, Stoller JR, Schulz RA (2009) New hemocyte-specific enhancer-reporter transgenes for the analysis of hematopoiesis in *Drosophila* . Genesis 47: 771–774. 19830816 10.1002/dvg.20561

[29] Ferrandon D, Jung AC, Criqui MC, Lemaitre B, Uttenweiler-Joseph S, et al (1998) A drosomycin-GFP reporter transgene reveals a local immune response in *Drosophila* that is not dependent on the *Toll* pathway. EMBO J 17: 1217–1227. 9482719 10.1093/emboj/17.5.1217 PMC1170470

[30] Andres AJ, Thummel CS (1994) Methods for quantitative analysis of transcription in larvae and prepupae. In: Goldstein LSB, Fyrberg EA, editors. *Drosophila melanogaster*: Practical uses in cell and molecular biology. San Diego: Academic Press. pp. 565–573. 10.1016/s0091-679x(08)60932-2 7535884

[31] Valanne S, Myllymäki H, Kallio J, Schmid MR, Kleino A, et al (2010) Genome-wide RNA interference in *Drosophila* cells identifies G protein-coupled receptor kinase 2 as a conserved regulator of NF-κB signaling. J Immunol 184: 6188–6198. 20421637 10.4049/jimmunol.1000261

[32] Pearson AM, Baksa K, Rämet M, Protas M, McKee M, et al (2003) Identification of cytoskeletal regulatory proteins required for efficient phagocytosis in *Drosophila* . Microbes Infect 5: 815–824. 12919849 10.1016/s1286-4579(03)00157-6

[33] Dunn OJ (1961) Multiple comparisons of means. J Am Stat Assoc 56: 52–64.

[34] Kroeger PT Jr, Tokusumi T, Schulz RA (2012) Transcriptional regulation of eater gene expression in *Drosophila* blood cells. Genesis 50: 41–49. 21809435 10.1002/dvg.20787

[35] Schlenke TA, Morales J, Govind S, Clark AG (2007) Contrasting infection strategies in generalist and specialist wasp parasitoids of Drosophila melanogaster. PLoS Pathog 3: 1486–1501. 17967061 10.1371/journal.ppat.0030158 PMC2042021

[36] Lanot R, Zachary D, Holder F, Meister M (2001) Postembryonic hematopoiesis in *Drosophila* . Dev Biol 230: 243–257. 11161576 10.1006/dbio.2000.0123

[37] Colinet D, Dubuffet A, Cazes D, Moreau S, Drezen JM, et al (2009) A serpin from the parasitoid wasp *Leptopilina boulardi* targets the *Drosophila* phenoloxidase cascade. Dev Comp Immunol 33: 681–689. 19109990 10.1016/j.dci.2008.11.013

[38] Gueguen G, Kalamarz ME, Ramroop J, Uribe J, Govind S (2013) Polydnaviral ankyrin proteins aid parasitic wasp survival by coordinate and selective inhibition of hematopoietic and immune NF-kappa B signaling in insect hosts. PLoS Pathog 9: e1003580. 24009508 10.1371/journal.ppat.1003580 PMC3757122

[39] Irving P, Ubeda JM, Doucet D, Troxler L, Lagueux M, et al (2005) New insights into *Drosophila* larval haemocyte functions through genome-wide analysis. Cell Microbiol 7: 335–350. 15679837 10.1111/j.1462-5822.2004.00462.x

[40] Shia AK, Glittenberg M, Thompson G, Weber AN, Reichhart JM, et al (2009) Toll-dependent antimicrobial responses in *Drosophila* larval fat body require Spätzle secreted by haemocytes. J Cell Sci 122: 4505–4515. 19934223 10.1242/jcs.049155 PMC2787462

[41] Parisi F, Stefanatos RK, Strathdee K, Yu Y, Vidal M (2014) Transformed epithelia trigger non-tissue-autonomous tumor suppressor response by adipocytes via activation of Toll and Eiger/TNF signaling. Cell Rep 6: 855–867. 24582964 10.1016/j.celrep.2014.01.039

