# Supplementary material for: Control of Drosophila Blood Cell Activation via Toll Signaling in the Fat Body
Source: PLoS One. 2014 Aug 7;9(8):e102568. doi: 10.1371/journal.pone.0102568 (PMC4125153; doi:10.1371/journal.pone.0102568)
Supplement: Figure S1 — Tissue specificity of the FB and Hml drivers. The expression of the FB-Gal4 (A–D) and HmlΔ-Gal4 (E–J) drivers was visualized by GFP fluorescence after crossing to the UAS-GFP reporter. Similar patterns were seen in Toll wild-type (A–B, E–G) and Toll10b genetic background (C–D, H–J). Panels A, C, C′, E and H show whole-body images of the GFP fluorescence. The demarcated areas in E and H are shown enlarged in panels F and I (bright-field images to the right). The fat body morphology in many, but not all Toll10b larvae is partially disrupted (compare C and C′). Panels B, D, G and J show hemolymph samples, in each case visualized by GFP fluorescence (GFP), Hoechst fluorescence (H) and differential interference contrast (DIC). Lamellocytes are marked by white arrowheads. (PDF) [file pone.0102568.s001.pdf]

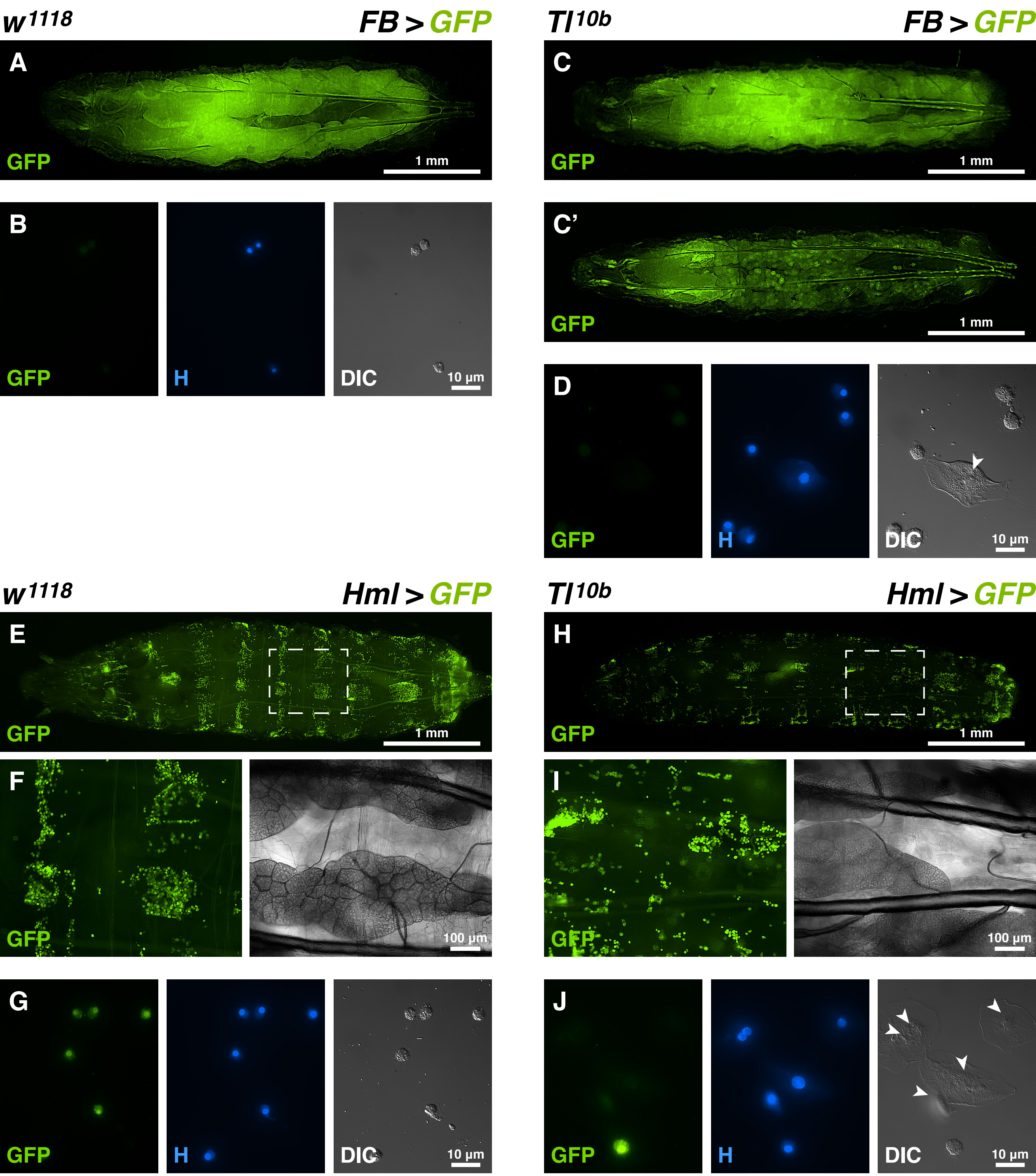

Figure S1. Tissue specificity of the *FB* and *Hml* drivers. The expression of the *FB-Gal4* (**A-D**) and Hml^{Δ}-*Gal4* (**E-J**) drivers was visualized by GFP fluorescence after crossing to the *UAS-GFP* reporter. Similar patterns were seen in *Toll* wild-type (A-B, E-G) and $Toll^{10b}$ genetic background (C-D, H-J). Panels A, C, C’, E and H show whole-body images of the GFP fluorescence. The demarcated areas in E and H are shown enlarged in panels F and I (bright-field images to the right). The fat body morphology in many, but not all $Toll^{10b}$ larvae is partially disrupted (compare C and C’). Panels B, D, G and J show hemolymph samples, in each case visualized by GFP fluorescence (GFP), Hoechst fluorescence (H) and differential interference contrast (DIC). Lamellocytes are marked by white arrowheads.
