# Supplementary material for: Control of Drosophila Blood Cell Activation via Toll Signaling in the Fat Body
Source: PLoS One. 2014 Aug 7;9(8):e102568. doi: 10.1371/journal.pone.0102568 (PMC4125153; doi:10.1371/journal.pone.0102568)
Supplement: Figure S2 — No effect of Toll signaling on the phagocytosis of bacteria. Toll signaling was activated by expression of UAS-Toll10b (>Tl10b), or suppressed by expression of UAS-MyD88GD25399 (>MyD88IR), either in hemocytes by HmlΔ-Gal4 (Hml>), or in fat body by FB-Gal4 (FB>). As a control, the eater RNAi construct eaGD4301 (>eaterIR) was also tested, but gave little effect. Hemocytes from larvae with these genotypes were incubated with FITC-labeled E. coli, and the phagocytosed bacteria are visualized by fluorescence, after quenching of extracellular bacteria with trypan blue. The top rows show controls without drivers. The leftmost panels show the drivers alone. (PDF) [file pone.0102568.s002.pdf]

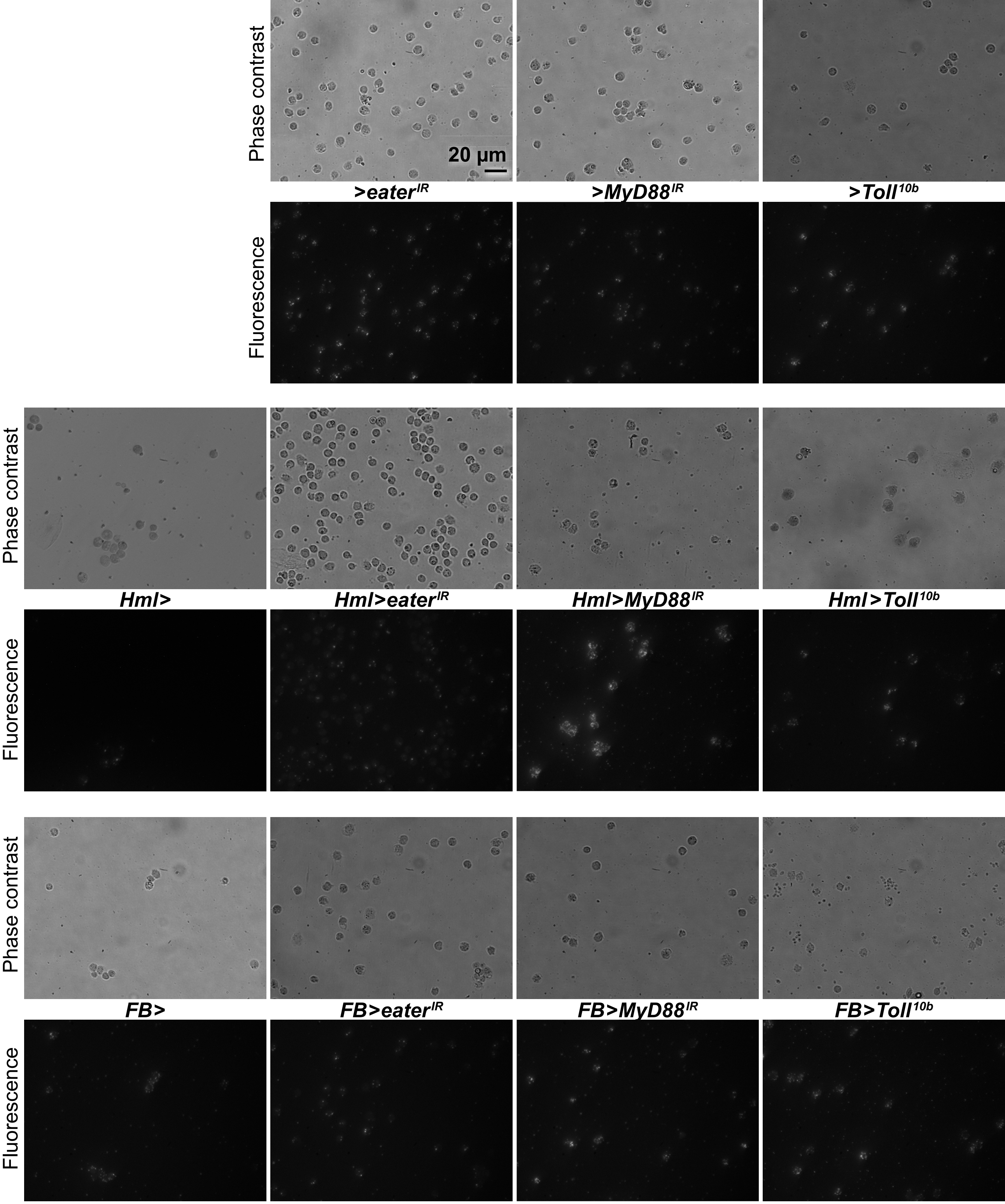

Figure S2. No effect of Toll signaling on the phagocytosis of bacteria. Toll signaling was activated by expression of *UAS-Toll*^{10b} (>*Tl*^{10b}), or suppressed by expression of *UAS-MyD88*^{GD25399} (>*MyD88*^{IR}), either in hemocytes by *Hml*$^{\Delta}$*-Gal4* (*Hml*>), or in fat body by *FB-Gal4* (*FB*>). As a control, the *eater* RNAi construct *ea*^{GD4301} (>*eater*^{IR}) was also tested, but gave little effect. Hemocytes from larvae with these genotypes were incubated with FITC-labeled *E. coli*, and the phagocytosed bacteria are visualized by fluorescence, after quenching of extracellular bacteria with trypan blue. The top rows show controls without drivers. The leftmost panels show the drivers alone.
